# Thaliacean tunicates, vertebrate sisters regained lifelong mobility

**DOI:** 10.1371/journal.pbio.3003674

**Published:** 2026-03-18

**Authors:** Yasunori Sasakura

**Affiliations:** Shimoda Marine Research Center, University of Tsukuba, Shimoda, Shizuoka, Japan

## Abstract

Tunicates are the closest living relatives of vertebrates, but many details of their evolutionary history remain unclear. This study explores the unique embryogenesis of Thaliacea, the tunicates that regained mobility from their sessile ancestor.

## Teaser

Tunicates are the closest living relatives of vertebrates, but many details of their evolutionary history remain unclear. A study published in *PLOS Biology* explores the unique embryogenesis of Thaliacea, the tunicates that regained mobility from their sessile ancestor.

Tunicates are the closest living relatives of vertebrates [1]; however, their morphology and lifestyle differ substantially from those of vertebrates. The sessile adult ascidians, the largest group of tunicates, most clearly exemplify this contrast. Their larvae are tadpole-like and actively swim by beating their tails [2]. The swimming larvae adhere to substrates and, through metamorphosis, lose their tails, undergo dramatic morphological changes, and enter a sessile life stage. Because the shared ancestor of tunicates and vertebrates is suspected to have been a free-swimming animal [1], the selective pressures that led ascidians to abandon mobility and evolve a sessile lifestyle remain central questions for understanding chordate evolution.

**Fig 1:**
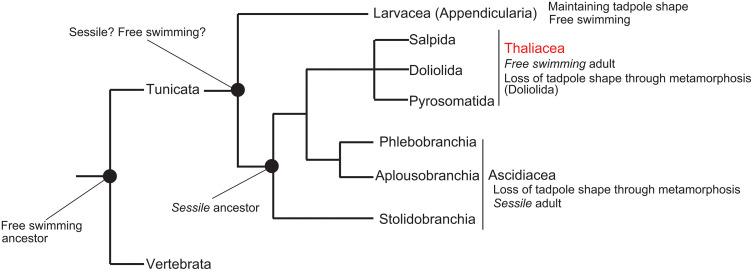
The phylogenetic relationship among tunicate groups, highlighting Thaliacea. This tree is according to the previous report [3]. Note that whether the shared ancestor of tunicates was a sessile or a free-swimming animal is under debate [10].

Thaliacea, a clade of tunicates, diverged from ascidians about 300 million years ago [3]. This phylogenetic position suggests that thaliaceans evolved from their sessile ancestor (Fig 1). However, they exhibit swimming behavior throughout their life cycle. Although adult thaliaceans possess a body shape similar to that of ascidians, they do not adhere to a substrate but instead swim by expelling seawater through their siphon. The emergence of thaliaceans is a mystery within the tunicate group: how a lineage that lost their mobile lifestyle regain this trait? Ecological factors exerted selective pressure that may have favored renewed locomotion [4]. The primary drivers of this unique evolutionary transition remain unknown. The swimming style of adult thaliaceans is different from that of vertebrate tadpoles and tunicate larvae. This means that thaliaceans regained their mobility not by maintaining a larval tail, as do larvaceans, but by modifying the developmental programs that construct and regulate post-metamorphic bodies. Perhaps the ancestral ascidian’s migratory mode was insufficient for the direct ancestor of thaliacean tunicates.

To elucidate the emergence of thaliacean tunicates, it is essential to characterize the cellular and molecular mechanisms underlying the development of extant thaliacean species through the application of modern research techniques. However, studies of this animal group are limited, likely because of the difficulty of collecting and maintaining or culturing laboratory samples. Historical studies are available for some thaliacean tunicates, but they are often contradictory, contain low-resolution images, and exhibit limited intra- and interspecific reproducibility. Research foundations for this animal group are needed to enable systematic investigation.

The new study by Lebel and colleagues addresses this gap through a detailed characterization of embryogenesis and the application of molecular techniques in two thaliacean species, *Salpa fusiformis* and *Thalia democratica* [5]. Both species belong to the order Salpida. Salps are viviparous, with embryogenesis proceeding within a specialized maternal organ. Ascidians, particularly solitary species, release gametes into seawater before fertilization. Some compound ascidian species, which form clonal aggregates, also exhibit embryogenesis in the parental body, suggesting that the capacity for viviparity may derive from a shared ancestral condition. After careful observation using a confocal microscope and the construction of developmental tables, the authors confirmed that embryogenesis patterns differ markedly between *S. fusiformis* and *T. democratica*. The variations encompass cleavage patterns, the formation of maternal organs, and global embryonic morphology. This contrasts with ascidians, whose embryogenesis features highly rigid cleavage patterns, resulting in embryos with nearly identical shapes across species [6]. A similar degree of stereotypy is observed in larvaceans, despite their reduced cell numbers, suggesting the loss of the tunicate mode of embryogenesis in salps. At the 8-cell stage, the calymmocytes, which arise from the follicles, begin to invade the embryo, physically separating individual blastomeres. Following this stage, calymmocytes increasingly participate in morphogenesis, indicating a sustained and substantial contribution to embryonic morphogenesis. The manner of morphogenesis mediated by calymmocytes is not identical between the two species. Interestingly, ascidian follicle cells are attached to the chorion and are released together with the eggs, raising the possibility that maternal cellular involvement in embryogenesis represents a conserved feature of tunicates. However, the extent of the contribution appears to vary among tunicate groups.

Using TUNEL staining, the authors examined apoptosis, or programmed cell death, during embryogenesis. They found that apoptosis occurs mainly in the epithelia of the incubation chamber, another maternally supplied tissue. By contrast, cell death in the calymmocytes is limited to a subset of cells, suggesting that this cell population remains functionally involved in embryogenesis after removal of the incubation chamber. The longevity of calymmocytes led the authors to hypothesize that these cells play pivotal roles in the patterning of salp embryos. This hypothesis was examined by analyzing the expression of two key developmentally relevant genes, *Otx* and *Rar*. Both genes encode conserved transcription factors responsible for establishing the body axes, especially the anterior‒posterior axis. Surprisingly, these genes are predominantly expressed in calymmocytes, suggesting that these maternal cells play regulatory roles in establishing body axes. It is generally accepted that calymmocytes are ultimately eliminated from the bodies of developing salps [7]. Thus, their contributions may not be the direct formation of a post-embryonic structure, but rather the provision of signaling cues to blastomeres for establishing body axes, as evidenced by the expression of *Rar*, which encodes the receptor for the key morphogen retinoic acid.

This article revisits the embryonic processes of salp tunicates, a sister group of vertebrates. Their unique features are likely to attract researchers to investigate the cellular and molecular mechanisms underlying their development and to examine the evolutionary implications of their emergence from sessile ascidians. Recent advances in sequencing technologies have benefited non-model organisms. Genome and RNA-seq data have been published in some salp species [8,9]. These sequence data will be best utilized within an established research background, such as descriptions of developmental processes at cellular resolution, developmental timetables, and methods for investigating genetic mechanisms, which this study has provided. Future studies grounded in these foundations will reveal how organisms adapt their characteristics flexibly in response to their environments over evolutionary time.
